# Analysis of Movements and Behavior of Bighead Carps (*Hypophthalmichthys nobilis*) Considering Fish Passage Energetics in an Experimental Vertical Slot Fishway

**DOI:** 10.3390/ani12131725

**Published:** 2022-07-04

**Authors:** Junjun Tan, Zhenbiao Liu, Yu Wang, Yuanyang Wang, Senfan Ke, Xiaotao Shi

**Affiliations:** 1 Hubei International Science and Technology Cooperation Base of Fish Passage, China Three Gorges University, Yichang 443002, China; tanjunjun52@163.com (J.T.); ksfctgu@163.com (S.K.); 2 College of Hydraulic and Environmental Engineering, China Three Gorges University, Yichang 443002, China; 19186249682@139.com (Z.L.); 15233430563@139.com (Y.W.); wangyuanyang911@163.com (Y.W.); 3 Engineering Research Center of Eco-Environment in Three Gorges Reservoir Region, Ministry of Education, China Three Gorges University, Yichang 443002, China

**Keywords:** fishway, movement trajectories, drag force, energy expenditure

## Abstract

**Simple Summary:**

Hydraulic structures have modified the natural waterways, and they have inevitably blocked fish migration routes and reduced fish habitat. To mitigate the impact on fish, fish passages have been developed as an effective way to restore riverine connectivity. The design of fish passage facilities refers to both hydrodynamics and fish behavior, and the information on the energetic expenditure of fish can be used to identify movement zones that are suitable for fish migration. Thus, this paper intended to identify fish movement behavior in response to water flow field information by means of estimating the energetic expenditure using an IBM approach. The results demonstrated that the fish required more energy in high TKE zones, and they were therefore likely to utilize the low TKE zones. This study provides a reference for optimizing the design of fish passages, and the method can be applied to assess the efficiency of fish passages and other fish bypass structures.

**Abstract:**

An understanding of fish movement behavior in response to flow field variables is important for exploring the hydrodynamic strategies of fish in fish passages. In this paper, bighead carps were taken as an example. The fish movement behavior response to water flow field information by means of estimating the energetic expenditure using an IBM approach in an experimental fishway was investigated. Fish swimming velocity, drag force, and energy expenditure were analyzed in varied flow conditions related to hydraulic variables, including velocity (V), turbulent kinetic energy (TKE), and strain rate (SR). The result indicated that the fish will require more energy in high TKE zones. This study provides a reference for optimizing the design of fish passages and fisheries management. This method can be applied to assess the efficiency of fish bypass structures and conduct fish survival studies.

## 1. Introduction

Hydraulic structures such as dams, weirs, and other instream structures have modified the natural waterways and inevitably made ecosystem-wide changes in rivers, especially for fish that live in rivers [1,2]. Fish migration routes have been blocked and the habitat has been reduced [3,4]. To mitigate the impact on fish, fish passages such as fishways or fish ladders have been developed as an effective action for restoring riverine connectivity [4,5].

The design of fish passage facilities involves both biologists and engineers. Engineers structurally design and improve fish passage facilities using physical models or computational fluid dynamics (CFD) [6,7,8,9,10]. Biologists have acquired fish movement behavior such as trajectories, energetic costs, etc., when fish are migrating [5]. Thus, it is important to track fish movement behavior and determine their hydrodynamic strategies.

The hydraulics of the fishway can be described by the Eulerian framework, while fish movement trajectories or movement behavior can be depicted by the Lagrangian framework, which can track and describe them as “particles”. An approach that combines the above two frameworks is therefore needed. An individual-based model (IBM) [11,12,13] approach can be applied to represent the interaction between fish swimming behavior and hydrodynamic conditions. One advantage of the individual-based framework is its relevance to internal states such as environmental conditions or individual heterogeneity [14,15]. Thus, an IBM approach was applied in this paper.

When considering the interaction between fish swimming behavior and hydrodynamic conditions, energy expenditure is a key indicator [16,17,18]. Guiny et al. [19] evaluated the fish energy expenditure when passing through different types of fishways such as weirs, slots, etc. Khan [17] used this model to estimate the drag force and energetic requirements based on the assumption that the fish had a constant swimming speed and there were three specified paths for adult salmon in a vertical slot fishway. However, fish behavior included passive, actively swimming against the flow, and actively swimming with the flow [5]. From a fisheries management perspective, information on site-specific energetic expenditure is critical for identifying zones that may prove difficult for fish migration. Therefore, this paper intended to present fish movement behavior in response to water flow field information by means of estimating the energetic expenditure in the process of fish’s actual movement from an IBM approach.

Carps, including black carp (*Mylopharyngodon piceus*), grass carp (*Ctenopharyngodon idella*), silver carp (*Hypophthalmichthys molitrix*) and bighead carp (*Hypophthalmichthys nobilis*) are typical potamodromous fish. They are the most commercially important freshwater fish species in China, especially in the Yangtze River basin. Carps, both adults and juveniles, undergo extensive spawning and feeding migrations [20]. Fish migrating upstream typically have an energy reserve to complete their migration [17,21]. Therefore, in this paper, the water velocity, which was exported from the CFD model, and the swimming speed of one of the four Asian carp species (bighead carp) were used to evaluate the drag forces and energy expenditure for analyzing the fish behavior selection in the fish passage. Specifically, the primary objectives of this paper were to (1) quantify the hydrodynamic strategies of the fish, and (2) determine the energetic expenditure for fish trajectories in a fishway.

## 2. Materials and Methods

### 2.1. Experiments

**Figure 1:**
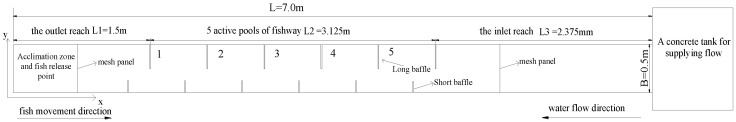
The planform of the experimental vertical slot fishway.

Laboratory experiments were performed in a 1:2.5 scale fishway model at the Hydraulics and Environment Department of the Engineering Research Center of Eco-Environment in the Three Gorges Reservoir Region, Ministry of Education, in China. It was 7 m in length with a height of 0.7 m, and it was externally reinforced by glass sidewalls (Figure 1). The inlet reach of the fishway was 2.375 m long, located at the upstream of the flume. The fishway consisted of five pools with a length of 0.625 m, height of 0.7 m and width of 0.5 m. The long baffles were 0.25 m and short baffles were 0.125 m; they were in a staggered arrangement on opposite sides of the sidewall. The fishway was set at a slope of 1%. An acclimation zone (0.7 m × 0.5 m × 0.7 m) was constructed 0.8 m downstream of the first pool (Tan et al., 2017).

### 2.2. Hydraulics

An acoustic Doppler Velocimeter (ADV) was used to measure the instantaneous velocity of the fishway. Considering that the hydraulic conditions (flow pattern, head drop) were similar in all pools, the measurements were carried out on the horizontal plane parallel to the flume bottom at a depth of Z = 0.3 h, 0.5 h, 0.7 h (h is the pool mean water depth) in the third pool. The experimental conditions are given in Table 1a. The hydraulics monitoring details can be found in a previous study [17]. The hydraulic variables such as velocity (V), turbulent kinetic energy (TKE) and strain rate (SR) were obtained.

Strain rate (SR) is a quantity that measures the degree of twisting, stretching, compression, and other forms of deformation on an element of water [22], which is defined as:

(1) $${\mathrm{SR}}_{\mathrm{ij}}=(\frac{\partial u_{i}}{\partial x_{j}}+\frac{\partial u_{j}}{\partial x_{i}})/2$$

TKE is the related velocity fluctuation at some point, which is defined as:

(2) $$\mathrm{TKE}\;=\frac{1}{2}\left(u_{x}{{}^{\prime }}^{2}+u_{y}{{}^{\prime }}^{2}+u_{z}{{}^{\prime }}^{2}\right)$$

(3) $$u_{i}^{\prime }=u_{i}-\bar{u_{i}}$$

where $u_{i}$ is the instantaneous velocity, $u_{i}^{\prime }$ is the fluctuating component of velocity at sampling time t, $\bar{u_{i}}$ is the mean velocity at the point, and $u_{x}^{\prime }$, $u_{y}^{\prime }$, $u_{z}^{\prime }$ are metrics of fluctuating velocity in the direction of x, y, and z.

### 2.3. Fish and Fish Trajectories

All tested bighead carps (N = 54), with a total length ranging from 10–15 cm (the details of the tested bighead carps are shown in Table 1b), were obtained from the Yidu hatchery during the migratory season. All fish were kept in a 1.8 m (diameter) × 0.3 m (height) × 1.5 m (height) aerated fish tank. In this paper, fish trajectories were obtained and analyzed under the discharge of 18.0 L/s. When starting an experiment, the mesh panel in the outlet section was removed to allow the fish’s voluntary movement in the fishway. If the fish could ascend into pool 5, the test was ended. Otherwise, the test continued for 1.5 h. After the experiments, all tested fish were returned to the tank. During the test, the movement trajectories of fish were continuously recorded by a video recording system. Logger Pro software was used to track the fish’s movement trajectories to determine the position of the fish.

Based on experimental monitoring and video observations, carps have a clear tendency to move close to the bottom of the fishway. Therefore, this paper mainly collected the fish movement trajectories and hydraulics data at z = 0.3 h in the xoy plane in all experiments.

### 2.4. Analysis of Fish Movement Using an IBM Approach

#### 2.4.1. Hydrodynamic Model

The hydraulics calculations for the fishway were carried out by computational fluid dynamics (CFD), as shown in a previous study [23]. It was widely considered that the flow field was a two-dimensional flow in the fishway with a small slope. Therefore, we obtained the xoy plane hydraulic distribution of the whole fishway from the hydrodynamic model. Hydraulic variables V, TKE and SR were obtained by the numerical simulation in the fishway.

#### 2.4.2. Fish Movement Model

Fish migrating upstream through the fishway would encounter the flow field and need to expend energy. Thus, the energetic expenditure of the fish during migration was analyzed. Because the fish movement trajectories were obtained at z = 0.3 h in the xoy plane, the model assumed that the fish movement trajectories were mainly in this plane and the fish adopted a non-jumping method (no ascending or descending in the vertical direction) of movement in the model.

In this model, we did not attempt to model muscular contractions and the subsequent effect on the surrounding water. The fish were regarded as particles, and Lagrangian particle-tracking was used to obtain the energy expenditure of fish in the migration. The swimming direction and swimming speed were based on the fish’s actual values. The flow variables, including V, TKE and SR, elicited the fish movement behavioral response in the energy model.

The fish swimming velocity can be represented as:

(4) $$\frac{\mathrm{dx}}{dt}=U(x)$$

(5) $$\frac{\mathrm{dy}}{dt}=U(y)$$

where the velocity U(*x*) and velocity U(*y*) are the velocity in the x and y direction, and x and y are the length of movement of individual fish in the x and y direction.

The hydrodynamic force acting on the fish was mainly drag force [24,25]. The expression to calculate the drag force [24,25] of fish in the process of movement in water is:

(6) $$f=0.5C_{d}\rho A_{s}{\left(U_{w}-U_{f}\right)}^{2}$$

where $C_{d}$ is the drag coefficient, ρ is the density of water, $A_{s}$ is the wetted surfaced area of fish, $U_{w}$ is the velocity of flow, and $U_{f}$ is the swimming velocity of fish. $C_{d}$ is the drag coefficient, which is composed of the frictional drag coefficient $C_{f}$ and the pressure drag coefficient $C_{p}$. In general, $C_{p}$ is smaller and can be expressed by the following formula according to the study of Pettersson and Bronmark [26]:

(7) $$C_{d}=C_{f}+C_{p}\approx 1.2C_{f}$$

C*_f_* and A*_s_* can be calculated as:

(8) Cf=0.074Ref−0.2

(9) $$A_{s}=\alpha L_{f}{}^{\beta }$$

where Re is the Reynold’s number, *α* and *β* are empirical coefficients, *α* = 0.465 and *β* = 2.11, and L*_f_* is the fish length [13].

Webb et al. (1984) [27] reported that the Reynold’s number of fish can be expressed as:

(10) Ref=ρufLf/μ

where μ is the dynamic viscosity of water.

The energy expenditure *E* of fish can be represented as following:

(11) $$E=\int_{0}^{s}\left|f\right|ds$$

where *s* is the length of fish trajectory [13,27]. In this study, energetic expenditure was related to the length of the fish (*L_f_*), the swimming speed (*U_f_*), and the movement path (*S*). 

### 2.5. Data Analysis

In order to analyze how water hydraulic parameters affect fish movement, the movement trajectories, swimming speed, drag force and energy expenditure were analyzed in different hydraulic conditions. The hydraulic variables V, TKE and SR were obtained from the CFD model results in a previous study [23].

## 3. Results

### 3.1. Hydraulics

**Figure 2:**
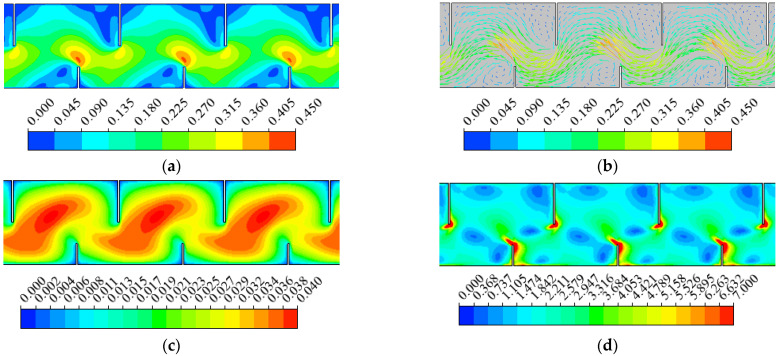
The hydraulics distribution: (**a**) velocity (m/s), (**b**) vector (m/s), (**c**) turbulence kinetic energy (m^2^/s^2^) and (**d**) strain rate (s^−1^) in pool 2–4 at z = 0.3 h, respectively.

The velocity and vector in pool 3 in the horizontal plane at z = 0.3 h are shown in Figure 2a,b. The velocity in the jet zone with maximum velocities was 0.51 m/s near the short baffles; the water flows sprayed through the upstream slot and then diffused in the pool. Two recirculation zones with velocities of 0.13–0.27 m/s were developed and characterized by low velocities on both sides of the jet zone.

The distribution of TKE and SR at z = 0.3 h are presented in Figure 2c,d. The SR was distributed along the mainstream flow so that SR was relatively large near the short baffles in the pool. The maximum value of SR reached 10 s^−1^.

### 3.2. Fish Movement Trajectories

**Figure 3:**
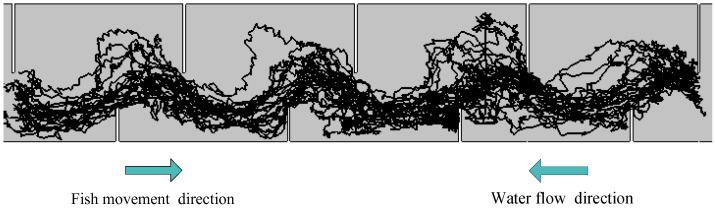
The typical movement trajectories of carps in pool 2–5.

The movement trajectories in the fishway are displayed in Figure 3. In total, 28 individuals’ trajectories were obtained. Of the successfully migrated individuals, some fish selected a short distance route, while some fish were inclined to move along the short baffle to choose a longer route.

**Figure 4:**
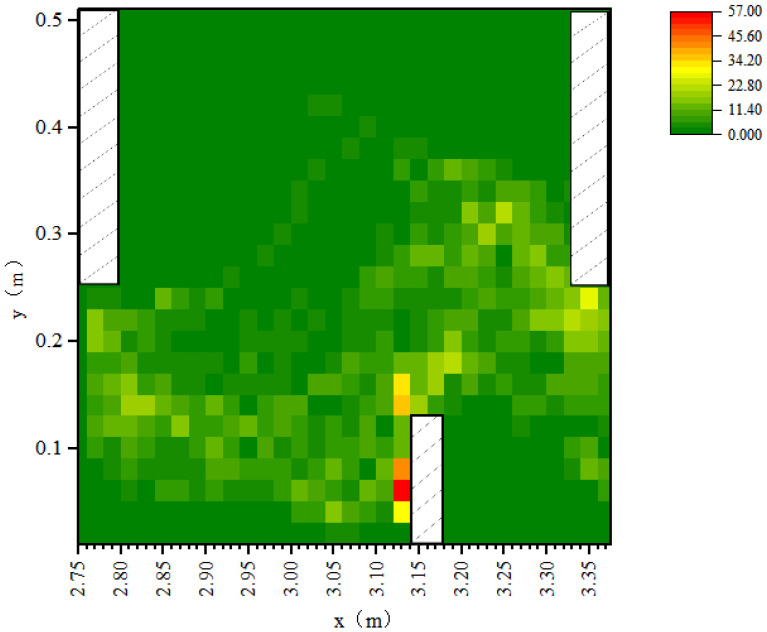
Heat maps of the fish occurrence number and movement trajectories with Q = 13.5 L/s. Note: the *x*-axis represents the coordinate value of pool 3 along the x direction, the *y*-axis represents the coordinate value of pool 3 along the y direction.

Because the movement trajectories were affected by hydraulic conditions, the hydraulic conditions that acted on fish movement were analyzed. Considering that flow was fully established in pool 3 and the hydraulic conditions in the other pools were similar to this one, the effect of hydraulics on fish movement was mainly analyzed in pool 3. The heat map of the fish occurrence number in pool 3 is shown in Figure 4. The fish were found to spend more time in this pool with the mean values ranging from 0.16–0.4 m/s, and the fish preferred regions with a mean TKE in the range of 0.020–0.036 m^2^/s^2^ and mean SR in the range of 2–7 s^−1^.

### 3.3. Fish Movement Energetic Expenditure

**Figure 5:**
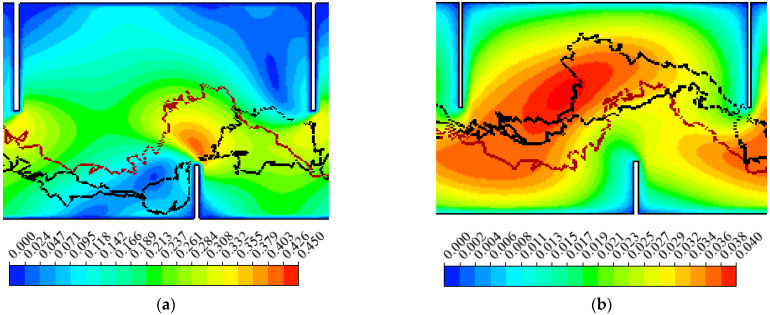

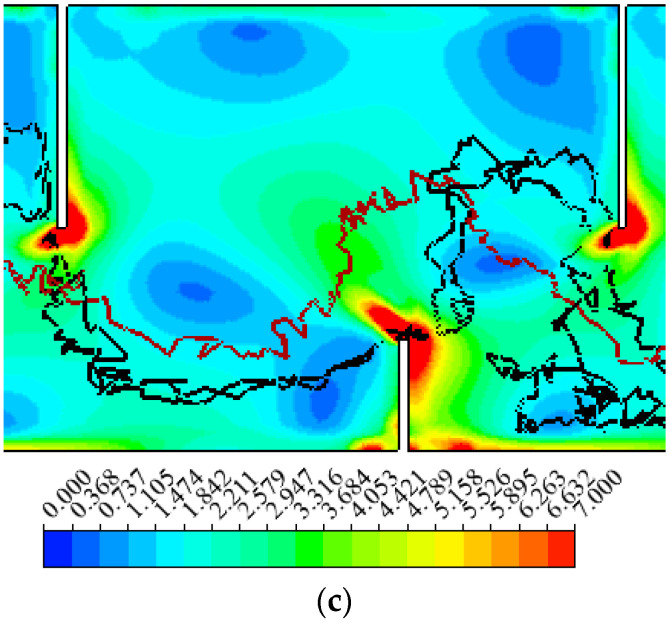
The movement trajectories of fish passing through typical hydraulics conditions: (**a**) typical 1: high velocity zone, velocity (m/s), (**b**) typical 2: high turbulence kinetic energy (TKE) zone, (m^2^/s^2^), (**c**) typical 3: high SR zone (Note: the black lines represent the fish trajectories passing through high V, TKE and SR zones, and the red line represents the fish trajectories without passing through the above-mentioned high zones).

In order to further analyze the effect of hydrodynamics on the fish’s movement, the drag force and the energy expenditure of fish under three typical hydraulics conditions were analyzed. Figure 5a shows the typical fish movement trajectories when passing through a high velocity zone near the slots (typical 1). Figure 5b shows fish passing through the high TKE zone (TKE was greater than 0.04 m^2^/s^2^) (typical 2). The movement trajectories of individual fish were affected by the high SR zone (greater than 6.0 s^−1^) near the long baffle, which is shown in Figure 5c (typical 3).

**Figure 6:**
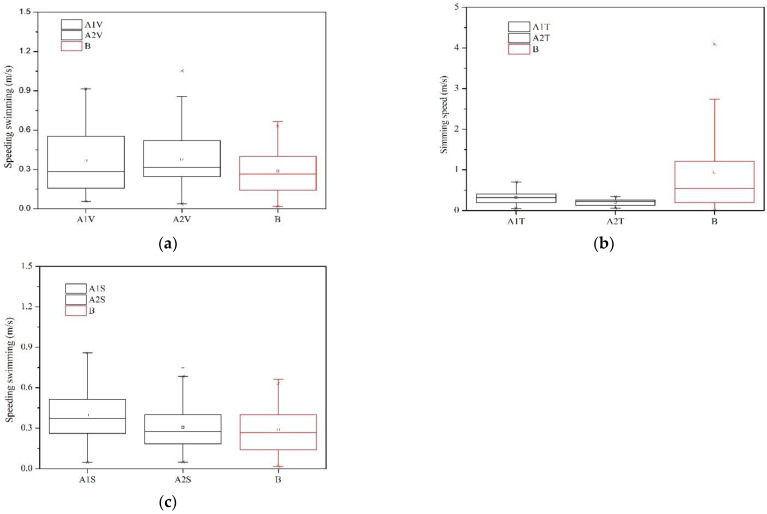
The magnitude of fish swimming speed under typical hydraulic conditions: (**a**) high velocity zone, (**b**) high TKE zone, and (**c**) high SR zone (Note: the black trajectories represent fish passing through high V (trajectories A1V, A2V), TKE (trajectories A1T, A2T) and SR (trajectories A1S, A2S) zones, the red trajectory represents fish not passing through the above-mentioned high zones).

**Figure 7:**
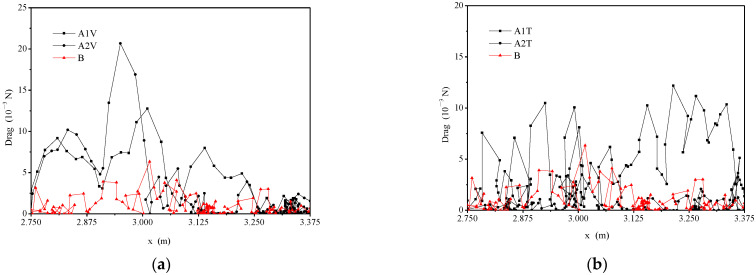

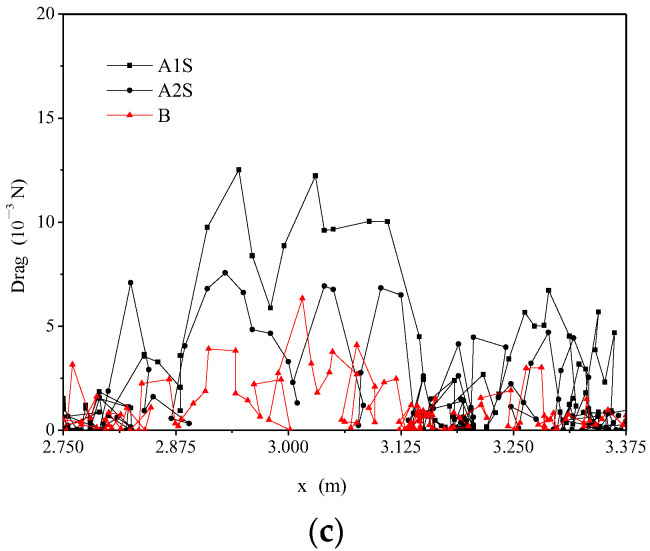
The drag force and the magnitude of fish under three typical hydraulic conditions: (**a**) high velocity zone, (**b**) high TKE zone and (**c**) high SR zone (Note: the black trajectories represent fish passing through high V (trajectories A1V, A2V), TKE (trajectories A1T, A2T) and SR (trajectories A1S, A2S) zones, the red trajectory represents fish not passing through the above-mentioned high zones).

**Figure 8:**
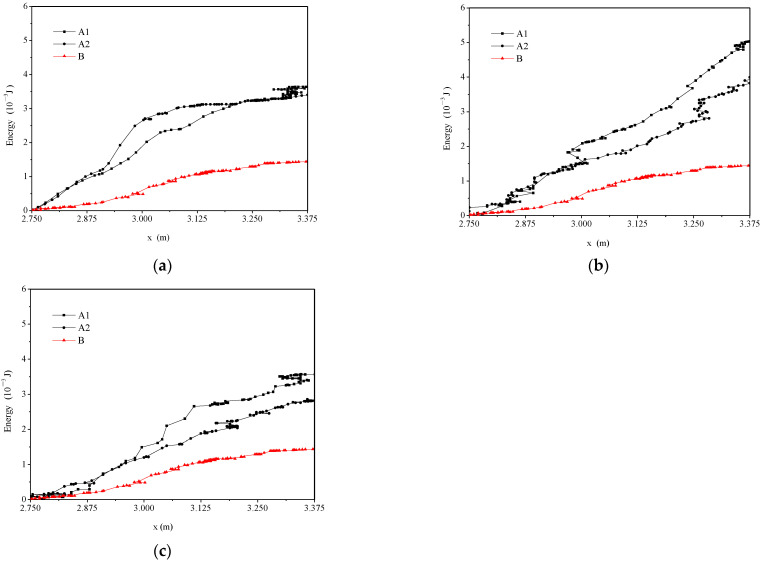
The cumulative energy expenditure of fish under three typical hydraulic conditions: (**a**) high velocity zone, (**b**) high TKE zone, and (**c**) high SR zone (Note: the x axis represents the coordinate value of pool 3 along the x direction, the black lines represent fish movement trajectories when passing through high V, TKE and SR zones, the red line represents fish movement trajectories without passing through the above-mentioned high zones).

Figure 6 shows the magnitude of fish swimming speed under three typical hydraulic conditions. The maximum swimming speed of fish was 1.054 m/s, 0.719 m/s and 0.859 m/s in typical 1, 2, 3, respectively. The average swimming velocity of fish ranged from 0.206 m/s to 0.397 m/s. Kruskal–Wallis tests on the swimming speeds for typical hydraulic conditions are shown in Table 2. Figure 7 shows the magnitude of the drag force under different hydraulic conditions. The drag force of typical 1, 2 and 3 was from 0 to 21.942 × 10^−3^ N. The maximum drag force was 21.942 × 10^−3^ N, 8.851 × 10^−3^ N and 12.51 × 10^−3^ N in typical 1, 2 and 3, respectively. The cumulative energy expenditure of fish under the three typical hydraulic conditions is shown in Figure 8. The average cumulative energy consumption required by fish was 3.521 × 10^−3^ J, 4.512 × 10^−3^ J, and 3.214 × 10^−3^ J when fish passed through the high V, TKE and SR zones in typical 1, 2, and 3. The fish movement parameters of typical 1, 2 and 3 are listed in Table 3.

## 4. Discussion

### 4.1. The Effects of Hydraulics on Fish’s Energy Expenditure

This study used an IBM approach to determine the energy expenditure of fish, which is different from the bioenergetics model used by Standen et al. [28] and Beauchamp et al. [29]. This approach can efficiently integrate hydrodynamic factors and the fish swimming speed, movement trajectories and drag force. In this paper, U*w* and U*_f_* were used as the actual velocity field values of the water and fish swimming speed. The measured movement trajectories played a significant role in calculating the drag coefficient and fish swimming energy expenditure. Therefore, the computational procedure presented in our study provided a relatively realistic estimate of fish energy expenditure.

In their natal river, anadromous fish migrating upstream typically have a finite energy reserve to complete their migration [21]. For the three hydraulic conditions, typical 1, 2 and 3, the average cumulative energy expenditure of typical 2 was the most, as shown in Figure 8b. Guiny et al. [19] suggested that movement in high turbulence zones imposed a high-energy cost. Enders et al. [30] showed that increased TKE resulted in an increased total swimming energetic expenditure for fish. In this research, the fish tended to have high energy expenditure when passing through high TKE zones during migration, which was consistent with the above-mentioned research. From another perspective, this revealed that TKE is a key hydraulic parameter that affects carps’ movement trajectories in a fishway.

Providing suitable swimming paths for fish is the ultimate aim of a fishway. Thus, it is critical to identify movement zones that are suitable for fish migration in terms of energetic expenditure. Due to the lack of physiological experiments, integrating the physiological indices to further understand the interaction between fish behavior and hydraulic metrics is future work. Considering that experimental bighead carps are not wild, the energy expenditure of these fish may be conservative and the conclusions may not be directly relevant to real fish passage. Even so, the approach can be used in the operation and management of fish passage, and it can be used in hydropower reservoirs and others fish bypass structures.

### 4.2. The Fish Swimming Strategies

The carp utilized cooperative and varied swimming strategies when passing through the water flow. Actually, the bighead carps took a longer transit time with a V range of 0.16–0.40 m/s, and most of the trajectories avoided the high TKE and SR zones and used the lower turbulence (0.02–0.036 m^2^/s^2^) and SR (2–7 s^−1^) zones when migrating upstream. Movement trajectories combined with video monitoring data showed that fish commonly passed through the zones of low TKE, with the exception of two fish that passed through the high TKE zone. Compared with the fish that did not pass through the high TKE zone, the fish appeared to have high transit times in the lower TKE zone (Table 3). However, in nature, fish are exposed to a complex water environment acting on their movement behavior [31]. The local velocity field would work on fish movement. Further study is required to create dynamic mesh methodologies for a CFD model to understand the complex field, such as nonhydrostatic free surface flows working on fish movement.

In this study, the carps’ drag force values calculated in this paper were less than 21.942 × 10^−3^ N. When the water thrust force (the reaction force of the individuals’ drag force) is less than 0.6 × 10^−3^ N, the fish is drifting with water, and when it is greater than 0.6 × 10^−3^ N, the fish are in accelerated motion [32]. Considering the magnitude of the drag force of fish under the three typical hydraulic conditions in Figure 7, it can be concluded that the fish were in motion most of the time, which was consistent with the observations and video monitoring. Additionally, the individuals’ trajectories were analyzed. Due to the assumption of non-jumping movement to calculate the energy expenditure of the fish movement trajectories, the carps’ energy expenditure could be under-estimated in this paper. Thus, obtaining the three-dimensional trajectories of fish for further accurate quantification of fish energy expenditure and movement strategy is future work.

It must be mentioned that a more actual mutual interaction between fish movement and water flow field was not considered in the current study. In this current study, the approach assumed a fish’s swimming direction with reference to the flow velocity in the xoy plane and more detailed behavior was not explicitly included in the paper, which would affect residence times and migration movement selection. The change in fish movement orientation could be a mechanism to increase the facticity of fish movement. Future research should be focused on the three-dimensional trajectories and the dynamic mesh methodologies of the CFD model to refine the results presented in this paper.

Although the approach used in this study has some limitations, it appears that this approach has potential merit for obtaining fish movement behavior while fish migrated in a fishway, while considering that the fishway was experimental and the conclusions have not been verified by field studies. In future, more refined work should allow for optimizing the structural design of the fish passage.

## 5. Conclusions

This paper presented fish movement in response to flow field information by means of estimating the energetic expenditure from an IBM approach. Combining the hydraulic variables (V, TKE and SR) and fish movement trajectories, the fish were found to spend more time, with mean V, TKE and SR values ranging from 0.16–0.4 m/s, 0.02–0.036 m^2^/s^2^ and 2–7 s^−1^, respectively, with a flow discharge of 13.5 L/s in a 1% fishway. The fish required more energy when passing through a high TKE zone. From an energy expenditure perspective, this revealed that TKE was a key hydraulic parameter that affects carp movement in a fishway, despite the fishway being experimental and the conclusions not being verified by field studies. In future, more refined work should be undertaken to optimize the design of fish passage structures.

## Figures and Tables

**Table 1:** (**a**) The experimental conditions for the test. (**b**) Details of tested fish: the total number of tested fish, total length and weight statistics for fish used in the experiments are shown.

(**a**)				
**Q(L/s)**	**H(m)**	**Slope (%)**	**Length of Pool (m)**	**Width of Pool (m)**
13.5	0.3	1	0.625	0.5
(**b**)				
**Fish**		**N**	**Total Length (cm)**	**Weight (g)**
bighead carp		54	11.41 ± 0.69	21.26 ± 3.63

Notes: (**a**) flow discharge (Q) and pool mean water depth (h).

**Table 2:** Kruskal–Wallis analysis on fish swimming speeds under different hydraulic conditions.

Index	Different Hydraulic Conditions	*p* Value
Swimming speed	TKE and V	*p* < 0.05
	TKE and SR	*p* < 0.05
	SR and V	*p >* 0.05

**Table 3:** (**a**) The fish movement parameters of typical 1. (**b**) The fish movement parameters of typical 2. (**c**) The fish movement parameters of typical 3.

Trajectories	Length of Trajectories (m)	Movement Time (s)	The Maximum Swimming Speed M (m/s)	The Average Swimming Speed (m/s)	The Average Drag (10^−3^ N)	The Maximum Drag Force (10^−3^ N)	The Cumulative Energy Consumption (10^−3^ J)
(**a**)							
A1 (Black line)	1.042	3.4	0.887	0.368	2.263	12.771	3.639
A2 (Black line)	0.842	3.28	1.054	0.375	2.295	20.675	3.403
B (Red line)	1.176	4.76	0.664	0.288	0.944	6.336	1.44
(**b**)							
A1 (Black line)	1.426	3.8	0.645	0.259	0.603	6.011	3.993
A2 (Black line)	1.159	4.72	0.719	0.323	1.449	8.851	5.03
B (Red line)	1.176	4.76	0.664	0.288	0.944	6.336	1.44
(**c**)							
A1 (Black line)	1.195	3.76	0.859	0.398	2.255	12.51	3.571
A2 (Black line)	1.516	4.8	0.684	0.301	1.429	7.563	2.857
B (Red line)	1.176	4.76	0.664	0.288	0.944	6.336	1.44

## Data Availability

The data presented in this study are available on request from the corresponding author.
